# Evolution of protein indels in plants, animals and fungi

**DOI:** 10.1186/1471-2148-13-140

**Published:** 2013-07-04

**Authors:** Pravech Ajawatanawong, Sandra L Baldauf

**Affiliations:** 1 Department of Systematic Biology, Evolutionary Biology Centre (EBC), Uppsala University, Uppsala 75236, Sweden

**Keywords:** Indels, Rare genomic changes, Phylogeny, Insertion/deletion, Multiple sequence alignment, Eukaryote evolution, Indel profiles

## Abstract

**Background:**

Insertions/deletions (indels) in protein sequences are useful as drug targets, protein structure predictors, species diagnostics and evolutionary markers. However there is limited understanding of indel evolutionary patterns. We sought to characterize indel patterns focusing first on the major groups of multicellular eukaryotes.

**Results:**

Comparisons of complete proteomes from a taxonically broad set of primarily Metazoa, Fungi and Viridiplantae yielded 299 substantial (>250aa) universal, single-copy (in-paralog only) proteins, from which 901 simple (present/absent) and 3,806 complex (multistate) indels were extracted. Simple indels are mostly small (1-7aa) with a most frequent size class of 1aa. However, even these simple looking indels show a surprisingly high level of hidden homoplasy (multiple independent origins). Among the apparently homoplasy-free simple indels, we identify 69 potential clade-defining indels (CDIs) that may warrant closer examination. CDIs show a very uneven taxonomic distribution among Viridiplante (13 CDIs), Fungi (40 CDIs), and Metazoa (0 CDIs). An examination of singleton indels shows an excess of insertions over deletions in nearly all examined taxa. This excess averages 2.31 overall, with a maximum observed value of 7.5 fold.

**Conclusions:**

We find considerable potential for identifying taxon-marker indels using an automated pipeline. However, it appears that simple indels in universal proteins are too rare and homoplasy-rich to be used for pure indel-based phylogeny. The excess of insertions over deletions seen in nearly every genome and major group examined maybe useful in defining more realistic gap penalties for sequence alignment. This bias also suggests that insertions in highly conserved proteins experience less purifying selection than do deletions.

## Background

While comparative studies of protein evolution focus mostly on conserved sequence blocks in multiple sequence alignments (MSAs), variable length regions and the insertion/deletions (indels) they harbor have provided unique insight into how proteins function [1-5] and evolve [6-8]. Indel studies have also led to the discovery of useful experimental [9] and drug targets [10,11], as well as powerful taxon diagnostics and phylogenetic markers [12-16]. However while DNA indels have been surveyed in depth to address specific evolutionary questions or characterize restricted taxon groups [17], there have been few recent attempts to systematically characterize protein indels broadly across eukaryotes or study their mode of evolution and phylogenetic distribution.

Early comparisons of protein sequences quickly established that indels in protein coding genes are mostly small, encoding 1–5 amino acids, and occur almost exclusively in loops linking structural elements at the solvent-exposed surfaces of protein structures [2,6,7]. This does not mean that indels are functionally unimportant. In fact, indels are more common in proteins that are “essential” [1], have relatively low sequence substitution rates [8] and are highly connected in protein interaction networks [3]. As components of surface exposed loops, indels are especially likely to be involved in intermolecular interactions and species-specific adaptations [2,18]. For example, strong positive selection for more and longer indels (5–8 times background) was demonstrated for an ion channel protein, resulting in changes in membrane depolarization rate and motility in sperm [9].

Much of the large scale study of indels has focused on improved structural modeling of protein loops often through indel databases such as LIP [19], ArchDB [20], PDBeFold [21], IndelPDB [22], SCINDEL [18], and IndelFR [4]. Analyses of these data have confirmed earlier findings that indels are commonly found in loops and turns [6,22], and established that indels and their boundaries have unique amino acid biases and elevated mutation rates [4,5]. Indel surveys have also been used to identify regions of the human genome under positive selection [23] and in the search for potential drug targets in human pathogens [11].

Indels have also long been considered of high potential value as phylogenetic markers [24,25]. This is because indels are generally more rare and less easily reversed than simple sequence substitutions, and indels are also considered to have a stronger impact on protein structure and function than single residue changes [24,25]. In fact, a number of important evolutionary hypotheses have been based on, or supported by indels [12,14,15,26-29]. Some researchers have even proposed quantitative analysis of large numbers of indels as an alternative to more conventional “sequence substitution” based phylogeny [30-33]. However, others have shown that indels are subject to the same systematic biases as substitution-based phylogeny, particularly hidden reversal (homoplasy), horizontal transfer [34-36], taxon sampling effects [37] and long branch attraction [37]. In addition, indels suffer from the problem of small numbers of characters, which exacerbates systematic artifacts [38].

Despite the structural, functional and phylogenetic importance of indels, their evolutionary patterns are still poorly understood. We sought to improve this situation using the substantial amount of sequence data now available from across eukaryotes, particularly from animals (Metazoa), green plants (Viridiplantae) and Fungi. To this end we identified a set of large (>250aa), universal and single copy (in-paralog only) eukaryotic protein orthologs. We then used our recently developed program SeqFIRE [39] to extract and classify all indels from a set of taxonomically broad multiple sequence alignments of these proteins. The indels in the resulting database were characterized in terms of various characteristics including size, complexity, host protein size, evolutionary pattern and phylogenetic distribution. These data reveal that insertions out-number deletions in these universal conservative proteins by an average of 2.31 to 1. The phylogenetic distribution of indels in these proteins is also found to be very uneven among and within the major groups of eukaryotes examined.

## Results

### Orthologous protein clusters from 35 proteomes

**Figure 1:**
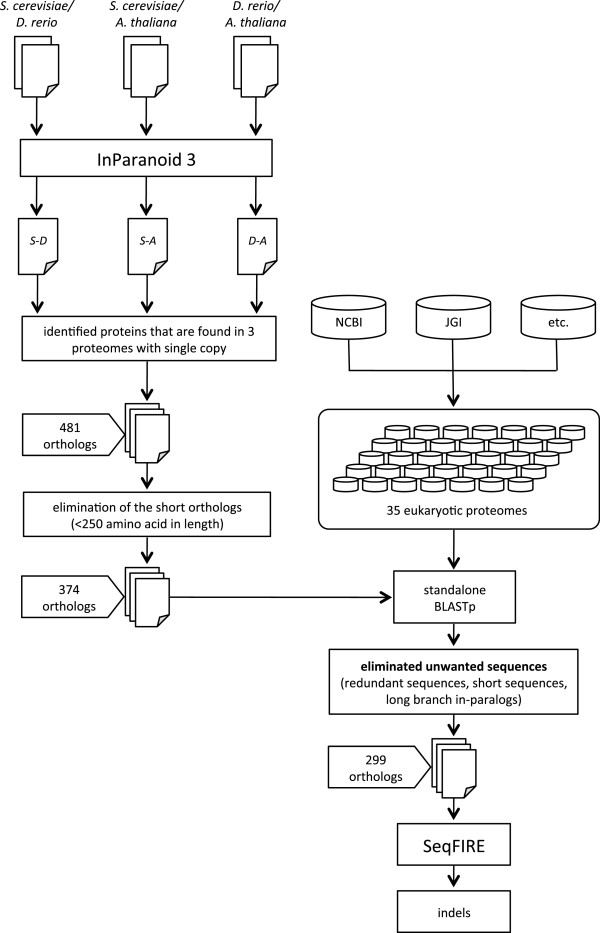
**Semi-automated pipeline for identifying universal eukaryote protein orthologs.** The diagram shows the workflow for identifying universal single or inparalog-only orthologous protein clusters. Orthologous protein candidates were identified using InParanoid version 3 [59] with pairwise comparisons among three starting test proteomes: *D. rerio*, *S. serevisiae*, and *A. thaliana*. The 477 orthologous protein candidates identified were used as seeds to BLASTp search 35 additional proteomes. The resulting putative orthologous clusters were aligned using MUSCLE version 3.6 [40,41], and screened by eye to eliminate incomplete sequences. Neighbor-Joining (NJ) trees were used to screen for redundant and unusually long branched sequences and to eliminate all but the shortest-branching sequence of each set of in-paralogs. Clusters found to include out-paralogs were partitioned into separate ortholog clusters. Clusters missing sequences from entire major taxa were also discarded. For the remaining protein alignments, indels were extracted using the program SeqFIRE. Genome combinations for the initial pairwise comparisons are indicated as follows: S-D (*S. cerevisiae* × *D*. *rerio*), S-A (*S*. *cerevisiae* × *A*. *thaliana*), D-A (*D*. *rerio* × *A*. *thaliana*).

**Figure 2:**
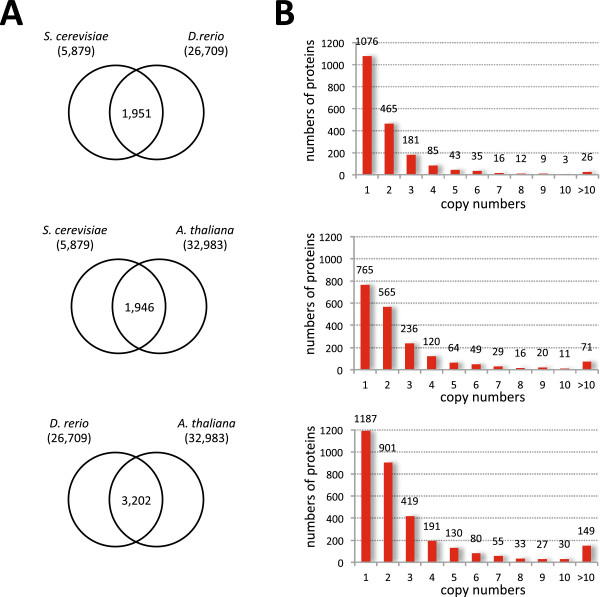
**Numbers and sizes of common orthologous protein clusters from pairwise comparison of three proteomes.** The set of common protein orthologs for three proteomes was identified by pair-wise comparisons of the proteomes using standalone InParanoid version 3.0 (panel **A**) [59]. The numbers of proteins in the orthologous clusters for each proteome pair are shown in bar charts (panel **B**). Genome combinations for pairwise comparisons are indicated as follows: S-D (*S. cerevisiae* × *D*. *rerio*), S-A (*S*. *cerevisiae* × *A*. *thaliana*), D-A (*D*. *rerio* × *A*. *thaliana*), and numbers of proteins in the individual proteomes are indicated in parentheses.

We conducted a broad survey of eukaryotic genome sequence data in order to identify substantial (>250aa), universal or nearly universal, single copy (out-paralog free) proteins (Figure 1) that could potentially be mined for evolutionarily interpretable indels. The protein size limit is required to provide sufficient phylogenetic information for meaningful control trees, which are needed to confirm sequence orthology. Emphasis was placed on well-sampled multicellular taxa, *i.e.*, plants, animals and fungi (Viridiplantae, Metazoa, Fungi) for which there exists a taxonomically broad genome sampling over which indel evolution can be traced. An initial set of seed orthologs was identified by pairwise comparison of the predicted proteomes of one representative each of Metazoa, Fungi and Viridiplantae (Figure 1). These were, respectively, *Danio rerio* (D), *Saccharomyces cerevisiae* (S) and *Arabidopsis thaliana* (A). Automated clustering of these proteomes predicted 1,951 (S-D), 1,946 (S-A) and 3,202 (D-A) orthologous protein clusters from the three possible pairwise combinations (Figure 2A). For each pairwise comparison, the largest fraction of clusters consisted of sequences that were single copy in both proteomes (Figure 2B), while the size distribution of the remaining clusters follows an exponential decay (Figure 2B). To reduce the chances of collecting multiple copies of orthologous proteins in further steps, only clusters that were single copy in this initial step were kept for further screening.

A total of 1,076 (S-D), 765 (S-A), and 1,187 (D-A) single copy orthologous protein pairs were identified by pairwise clustering (Figure 2A), of which 481 were found to be single copy in all three predicted proteomes. Of these 481 clusters, 107 were discarded because they consisted of proteins shorter than 250aa. All proteins in the 374 remaining clusters were then expanded to include data from 32 additional taxa, by BLASTp searches using all proteins in each cluster as query sequences against individual complete predicted proteomes (Figure 1). BLASTp results were filtered to remove redundant or incomplete sequences, and clusters with poor taxonomic representation were discarded (see Methods). Multiple sequence alignment and phylogenetic analysis were then used to select long-branched in-paralogs for removal. Clusters with universal out-paralogs (present in most or all taxa and forming a separate monophyletic group), which represent ancient gene duplications, were separated into unique clusters, which were then re-submitted to the pipeline. The final result was 299 unique clusters of substantial, universal single copy (or in-paralog only) orthologous proteins.

### Indel extraction protocol

**Figure 3:**
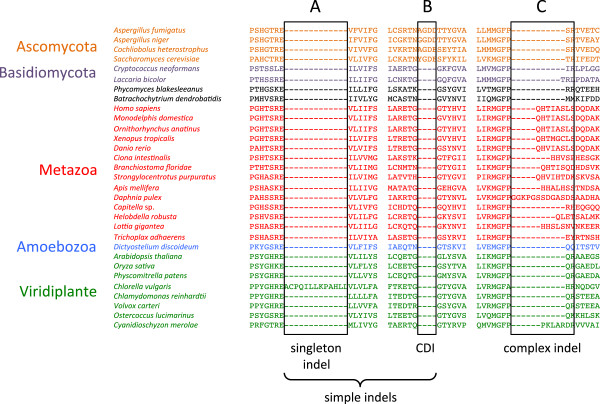
**Examples of simple and complex indels.** A partial protein sequence alignment of transcription initiation factor TFIIH subunit H2 from 31 eukaryotic species is shown. Two example of simple indels are shown in block **A** and **B**, and a complex indel is labeled as block **C**. Indel A is a singleton indel (found in only one sequence), and indel B is a multi-residue CDI (clade defining indel).

Each of the 299 universal orthologous protein clusters was re-aligned using MUSCLE [40,41] and then re-submitted to SeqFIRE for indel extraction [39]. SeqFIRE automatically extracts indels based on a set of user-defined criteria, the most important of which is the stringency (amino acid conservation threshold) of the guide consensus sequence. This guide determines which alignment columns will be identified as conserved, which is critical in determining indel boundaries. SeqFIRE also classifies indels into two different categories: “simple indels” occur in only two states, present or absent, and are potentially the result of a single indel event, while “complex indels” occur in two or more states and represent multiple indel events (Figure 3).

**Figure 4:**
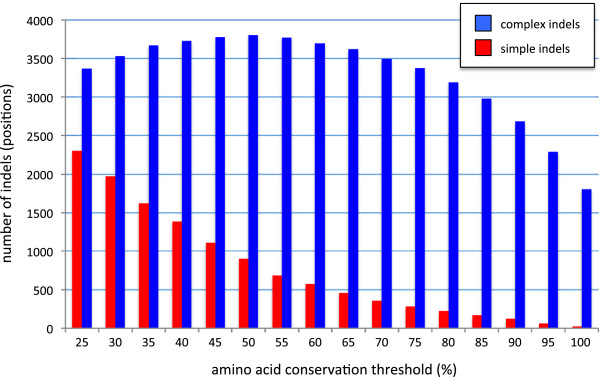
**Identifying an optimal sequence similarity threshold for indel extraction.** The vertical bars indicate the number of complex (blue bars) and simple indels (red bars) that were identified by SeqFIRE under a range of similarity threshold scores for the guide sequence.

In order to identify an optimal consensus level for indel identification, indels were extracted from the 299 alignments under increasing levels of stringency from 25% (the general minimum level observed for homologous proteins or “twilight zone” [42]) to 100%, in incremental steps of 5% (Figure 4). Low stringency results in many small indels, the majority of which are simple indels, while high stringency results in fewer but larger and mostly complex indels (Figure 4). This is because raising the stringency level causes fewer sites to be identified as conserved with the result that indels separated only by regions of low sequence conservation are merged, forming large complex indels instead. Thus the size distribution of simple indels shows an exponential decay with increasing similarity threshold (Figure 4, red bars), while that of complex indels shows a bell shaped size versus frequency curve (Figure 4, blue bars). In order to maximize the balance between stringency and sensitivity, we selected the peak of this curve (similarity level = 50%) as the optimum threshold for indel extraction. A total of 4,707 indels were then extracted using these optimized criteria, of which 901 (19.1%) were classified as simple indels and 3,806 (80.9%) were classified as complex indels.

### General characterization of indels

**Figure 5:**
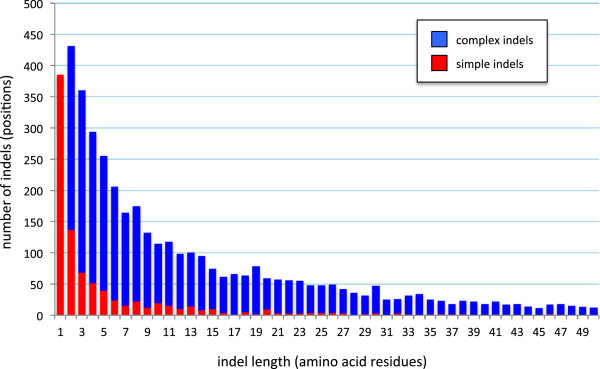
**Length distribution of eukaryotic protein indels.** For each indel size class (x-axis), the number of simple (total = 901) and complex (total = 3,806) indels are indicated by the red and blue bars, respectively. 501 indels (10 simple indels and 491 complex indels) longer than 50 amino acid residues are not shown.

Overall, the most frequent indel class is the single amino acid (1aa) indel, which by definition is always a simple indel. These 1aa indels account for 8.2% of all indels and nearly half (42.7%) of all simple indels (Figure 5). Simple indels in general are mostly short (85.7% are ≤10aa, 75.5% are ≤5aa), with a median length of 2aa, and simple indels larger than 15aa are extremely rare (Figure 5). Thus the pattern of length distribution of simple indels shows a steep exponential decay. Complex indels (mean length = 12aa) occur in a much wider size range than simple indels and have a much more gradual exponential decay with a much longer tail (Figure 5).

**Figure 6:**
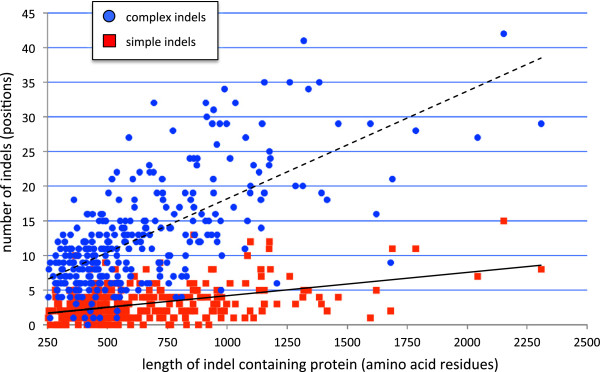
**Relationship between host protein length and number of indels.** Red squares and blue circles indicate the number of simple and complex indels, respectively, found in different length indel-host proteins. The solid line shows the regression line of simple indels (R^2^ = 0.2048), and the dashed line shows the regression line of complex indels (R^2^ = 0.4550). Proteins shorter than 250 amino acid residues were excluded from the analysis.

Previous work has shown that protein indel frequency but not indel size is correlated with protein length [7]. Since these findings were based on pairwise comparisons, which cannot distinguish between simple versus complex indels, we examined the relationship of indel frequency to protein size for our two different indel classes. Both simple and complex indels show a linear relationship between indel frequency and host protein length (Figure 6), although for simple indels the slope of the line is much lower (0.0034 versus 0.0155) as these indels are much more rare. This shows that there is only a small difference in simple indel frequency for proteins between 250 to 1,000 residues in length, which is the vast majority of proteins (Figure 6) [43]. Thus although the chances of finding complex indels increases substantially with host protein length, this trend is much weaker for simple indels.

**Figure 7:**
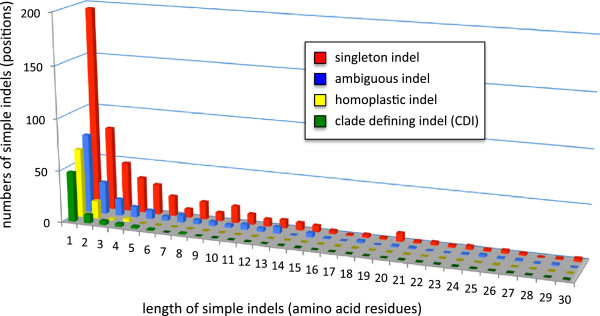
**Length distribution of simple indel types.** The entire database of simple indels extracted here (901 indels) was classified into 4 subclasses: singleton indels (red bars), clade defining indels (CDIs, green bars), homoplastic indels (yellow bars) and ambiguous indels (blue bars). The height of the bars shows the number of all simple indels (y-axis) of each type in each size category (x-axis).

We further examined the evolutionary patterns of simple indels by classifying them into three different types based on the fit of their distribution to accepted evolutionary relationships, which are well resolved for most of the species examined here. Type 1 or “singleton indels” are found in a single taxon only and thus appear to have arisen relatively recently on the evolutionary time scale examined here (Figure 7). Type 2 or potentially evolutionarily informative indels appear as universally shared by some taxa. Finally, type 3 or “ambiguous indels” are indels that were extracted from an alignment that lacked sequences from some sister-taxa and are therefore difficult to interpret with certainty (Figure 7). Of the 901 simple indels identified here, 550 (61%) are singletons, 195 (21.6%) are ambiguous and the remaining indels are potentially informative.

### Analysis of potentially evolutionarily informative indels

**Figure 8:**
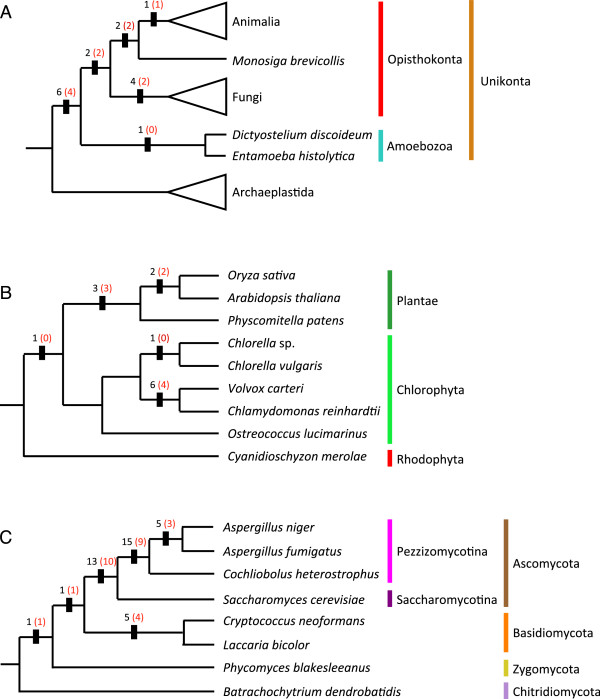
**Clade defining indels mapped onto simple consensus phylogenies of eukaryotes, Viridiplantae and Fungi.** Simple phylogenies of all species in the dataset **(A)**, for Archaeplastida **(B)** and for Fungi **(C)** were reconstructed from the literature [45,46]. The black numbers on the branches indicate the total number of informative indels for that particular branch, and the red numbers indicate how many of this total are single amino acid (1aa) indels.

Protein indels are widely considered to be powerful phylogenetic markers [26,44]. Therefore, we examined the potential for the indels described here to mark major events in eukaryote evolution. We further classified the 156 potentially evolutionarily informative indels by mapping them onto consensus phylogenies extracted from the literature [45,46]. This shows that 87 (55.8%) of these indels are in fact homoplastic, that is, they are present in two or more unrelated taxa, and therefore assumed to have arisen independently in each taxon. The remaining 69 (44.2%) indels are referred to here as “clade defining indels” (CDIs). These are indels that appear to be phylogenetically informative for the taxon set used here (Figure 7). At the deepest taxonomic level examined, a total of 16 indels are found that define the supergroup unikonta or major divisions within it (Figure 8A). Eleven of these apparently very ancient CDIs are 1aa indels, and the remaining five are multi-residue (>1aa) indels (Figure 8A). The six CDIs uniting unikonta and the two uniting Opisthokonta to the exclusion of Amoebozoa are particularly interesting, as they may be useful in resolving the phylogenetic position of enigmatic taxa currently assigned to this region of the tree, but unresolved within it, such as the single-celled Ancyromonads and Apusomonads [45].

Within the three major multicellular groups examined here, we find a very uneven distribution of CDIs. Only a single CDI is identified supporting the clade Metazoa and none were found supporting any represented groups within it (Figure 8A). This is not an artifact of the denser taxon sampling in Metazoa, as we do not find any potentially useful CDIs for Metazoa even among the discarded ambiguous or homoplastic indels. Meanwhile 13 CDIs were found for clades within Viridiplantae (Figure 8B) and 40 for clades in Fungi (Figure 8C). The lack of CDIs within Metazoa seems somewhat surprising as this is the single most widely sampled taxon here, including 15 genomes from representatives of the three major divisions (Deuterostomia, Ecdysozoa, and Lophotrochozoa) plus the enigmatic placozoan, *Trichoplax*[46]. Within Viridiplantae almost half of the CDIs are found in the relatively closely related chlamydomonads, *Volvox carteri* and *Chlamydomonas reinhardtii*. Nonetheless, three 1aa CDIs are found in land plants and absent from the other major examined clade of green algae, the “CUT” algae (Chlorophyta + Ulvophyta + Trebouxiophyta, Figure 8B). These CDIs could be potentially useful, for example in screening possible sister taxa to land plants.

In contrast to Viridiplantae and especially Metazoa, we recovered a total of 40 CDIs from Fungi (Figure 8A and 8C). Thirty of these CDIs are from the Ascomycota, including five that are uniquely shared by two species of *Aspergillus* that appear to be closely related [47]. Fifteen of these CDIs mark a deep clade of Ascomycota (Pezzizomycotina) excluding *Saccharomyces*, which appears to be a very early branch of Ascomycota [47]. Nearly half (9/15) of these are also >1aa CDIs. This suggests that protein indels could be a useful tool for fungal phylogeny or as diagnostics at a number of different taxonomic levels.

### Evolutionary patterns in singleton indels

**Figure 9:**
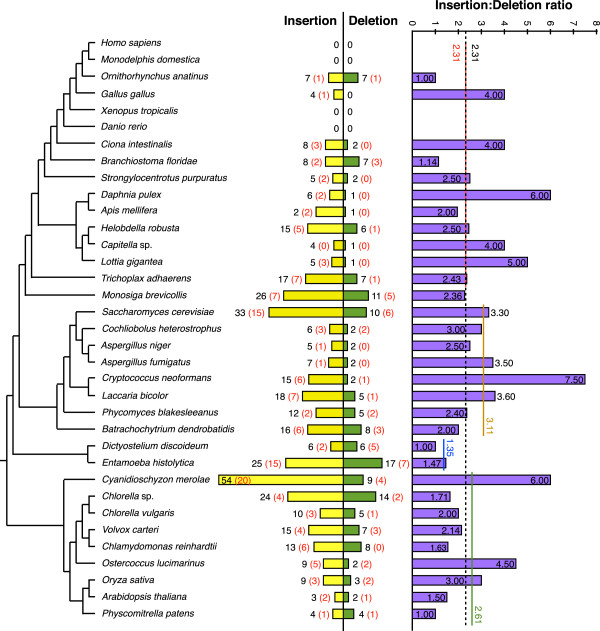
**Phylogenetic profile of singleton insertions and deletions.** The number of all (black) and 1aa (red) singleton insertions (yellow bars) and deletions (green bars) in universal orthologous proteins over 250 amino acids in length, are displayed on a schematic phylogeny of the 35 examined species. The insertion:deletion ratios of the singleton indels for each species on the tree are shown on the far right of the figure, within or beside the purple bars, which are drawn to scale as indicated by the scale bar at the top of the column. The dashed line shows the average insertion:deletion ratio for the full dataset, and the red, orange, blue and green lines show the insertion:deletion ratio of Metazoa, Fungi, Amoebozoa and Archaeplastida (Viridiplantae + Rhodophytae), respectively.

By far the largest fraction of indels we identify are indels that appear as singletons for the taxa examined here. These singleton indels total 550, constituting roughly two-thirds (61%) of all the simple indels identified, and these indels show a very erratic distribution across the phylogeny (Figure 9). Singletons are expected to be most common in poorly represented major taxa, as many of these indels would probably be redefined as CDIs or homoplastic indels with additional taxon sampling. Thus it is perhaps not entirely surprising that the sole red alga in our data set, *Cyanidioschyzon merolae*, shows the largest number of singleton indels (63 indels, Figure 9). However, other single representatives of ancient lineages show much lower numbers of singletons, such as *Monosiga,* the sole choanoflagellate, (37 singleton indels) or *Batrachochytrium*, the sole chytrid (24 singleton indels, Figure 9). The average number of singleton indels per examined genome is also considerably lower in Metazoa (116/15 = 7.7) than in Fungi (148/8=18.5) or green plants (132/8=16.5). Thus the frequency of indels in universal conserved proteins appears to vary widely among evolutionary lineages.

A total of 391 of the 550 singleton indels identified here are insertions, giving an average insertion:deletion (I:D) ratio of 2.31 (Figure 9). The average singleton I:D ratio is also fairly consistent among the three best sampled lineages - Metazoa, Fungi, and Archeaplastida (Viridiplantae + Rhodophyta), which exhibit singleton I:D ratios of 2.31, 3.11, and 2.61, respectively (Figure 9). Of the 31 taxa in which we find singleton indels, only eight show a singleton I:D ratio of less than 2.0 (Figure 9), and we find no example of a taxon with a singleton I:D ratio of less than 1.0. Thus we find no taxon for which deletions are more common than insertions for these proteins. Nonetheless, singleton I:D ratios can vary widely among individual taxa; we find 13 taxa for which the singleton I:D ratio is ≥3.0, of which eight taxa have a ratio ≥ 4.0 (Figure 9). Thus, despite a wide variation in singleton frequency, these indels show an almost universal bias toward insertions over deletions across a fairly broad taxonomic sampling of eukaryotes, (Figure 9).

We find no obvious pattern in singleton I:D ratios among these taxa. Taxa with high and low singleton I:D ratio are found scattered amongst each other and across the tree, and high and low ratios are found in both singleton-rich and singleton-poor taxa (Figure 9). Both high and low ratios are found in the four obligate parasites examined here, *Batrachochytrium, Cryptococcus, Cyanidioschyzon and Entamoeba*, which show singleton I:D ratios of 2.0, 6.0, 7.0 and 1.47, respectively (Figure 9). Nor does multicellularity appear to bias I:D ratios; Metazoa, which is represented here almost exclusively by multicellular taxa, has an average I:D ratio identical to the overall I:D ratio of 2.31. Metazoa also includes *Daphnia* (Crustaceae), which has one of the highest singleton I:D ratios (6.0), while its sister taxon *Apis* (Insecta) has one of the lowest (I:D = 1.0). Thus we find no obvious taxonomic or life-style pattern in singleton I:D ratios among the taxa and genes examined here.

**Figure 10:**
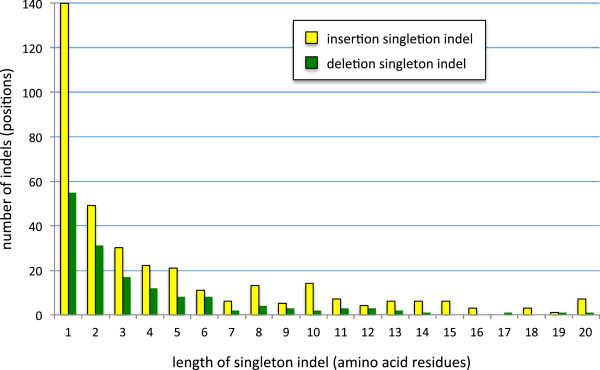
**Size distribution of singleton insertion and deletion indels.** The numbers of singleton insertions (yellow bars) and deletions (green bars) is shown for different indel sizes, on the y- and x-axes, respectively. Five deletions and 40 insertions larger than 20aa are not included.

The excess of insertions over deletions suggests that eukaryotic proteins should be increasing in size over time. However, previous comparisons across the three domains of life found no such trend [8]. Therefore we compared the size of insertions versus deletions in the singleton indels collected here. We find that insertions are more common than deletions in every single indel size class (Figure 10). Therefore eukaryotes have not avoided protein size increase by balancing many small insertions with fewer but larger deletions. Nonetheless, despite the large number of singleton indels we find, these are still very rare on the evolutionary time scale examined here. These insertions are also very small (over 50% are 1aa). Therefore, they are unlikely to have a significant impact on protein size.

## Discussion

We have analyzed 35 complete eukaryote proteomes and identified 901 universal single copy (or in-paralog only) orthologous proteins of substantial size (>250aa) (Figures 1 and 2). After determining an optimal consensus level of 50% for the guide sequence used to identify indel boundaries (Figure 4) [39], we found 4,707 indels in these proteins, of which 901 are classified as simple (binary-state) and 3,806 as complex (multi-state) (Figure 3). The majority of simple indels are found in only a single genome (singleton indels, 61%). However, we are still able to identify 69 apparently simple indels that mark major clades in eukaryote evolution and are therefore potentially useful as phylogenetic markers or diagnostics of these clades (Figure 8). Using singleton indels, we find that insertions are over twice as frequent as deletions, at least for this data set, which consists of universal conservative proteins, largely represented by Metazoa, Viridiplantae and Fungi (Figure 9).

### Indel size distribution

Our results are consistent with previous studies showing that the vast majority of protein indels are short [6,7,9,48]. This size limit is particularly strong for simple indels, 42.7% of which are 1aa in size and 37.1% of which are 2-7aa (Figure 5). Except for indels of the 1aa size class, which are always simple, complex indels outnumber simple indels in all size classes by a ratio that increases exponentially with size (Figure 5). Thus, larger indels are mostly complex. Although it is tempting to speculate that small simple indels grow into large complex ones by serial insertion events, this is inconsistent with the fact that indel length distribution is largely independent of evolutionary distance [7]. That suggests instead that there may be a qualitative difference between sites that tend to harbor large, mostly complex indels versus sites that tend to harbor small, often simple indels.

Although we confirm an exponential decay in indel frequency with increasing indel size, our data show a much slower decay, larger size spread and longer tail than previously reported [6,7], particularly for complex indels (Figure 5). This may be partly due to the much larger size and more comprehensive nature of currently available protein sequence data. However this difference is mostly due to the fact that we extract indels from MSAs, unlike previous studies that used pairwise alignment [6,7]. This causes nearby indels separated by poorly conserved sequence to be merged, resulting in the larger size spread and longer tail observed here (Figure 5). This distribution is also affected by the consensus threshold used for indel extraction (Figure 4), which was chosen here to maximize the balance between stringency and sensitivity and is thus a compromise between the two.

### Phylogenetic distribution of indels

Phylogenetically useful indels can be classified into two types – phylogenetically informative indels (CDIs), which are found in groups of related organisms (Figure 7), and singleton indels, which are unique to individual species (Figure 9). Thus CDIs are potentially diagnostic of taxon groups, while singletons are potentially diagnostic of single species. Given the abundance of singleton indels we find here (550 singletons extracted from 299 proteins), the potential for their use as taxon diagnostics appears to be substantial. However, this depends on taxon sampling, as many of the singletons identified here may occur throughout a taxon group for which we have sampled only one individual. Such an indel would then be a CDI for that group, rather than a singleton. Alternatively, denser taxon sampling of CDI indels may reveal homoplasy that has escaped detection with the limited taxon sampling we use here. This reinforces the point that all potential singleton indels and CDIs require further analysis with denser taxon sampling to test their utility with respect to specific phylogenetic questions.

While most simple indels are small (42.7% = 1aa, 15.2% = 2aa, Figure 5) and show a fairly high level of homoplasy (15.9%, Figure 7), 619 simple indels were found that appear to be homoplasy-free for the taxa examined here, and 69 (7.7%) of these vary among clades in a manner consistent with known phylogeny (Figure 8). Thus CDIs make up a small fraction (1.5%) but still a substantial number of these indels. Some of these CDIs mark major branches in eukaryotes and could be useful in assigning enigmatic taxa to the relevant clades. However, this potential is not the same for the three major taxon groups; Viridiplantae and especially Fungi are relatively rich in CDIs (13 and 40, respectively), while Metazoa have one (Figure 8). Thus there seems to be considerable potential for indels as clade diagnostics in Fungi and possibly also in green plants, but little potential for Metazoa, at least for these universal single copy proteins.

This lack of CDIs in Metazoa seems surprising, as we include nearly twice as many metazoan taxa in our analysis as we do for the other two multicellular groups, including substantial taxonomic breadth across Metazoa (Figures 8 and 9). While including more taxa increases the chances of discovering homoplasy and therefore ruling out possible CDIs, we do not find any potential CDIs for Metazoa even among the homoplastic and ambiguous indels we discard. Instead, this lack of metazoan CDIs is probably related to the fact that Metazoa have an unusually slow evolutionary rate for these universal conservative proteins. Using a 70% consensus of all universally aligned positions in our data set, we find that 71% of the consensus positions are conserved across Metazoa versus 60% for Viridiplantae and 58% for Fungi (Additional file 1: Table S1). Thus, a similar analysis of less conservative, perhaps even metazoan-specific proteins could be more productive in identifying CDIs for major clades within Metazoa. In addition, some indels identified here as homoplaseous across a wide sampling of eukaryotes may still be homoplasy-free for more restricted taxon sets such as, for example, Metazoa [49].

Nonetheless CDIs, at least by our strict definition and at the taxonomic depth examined here, appear to be too rare for quantitative phylogeny. Although it has been argued that such macromolecular characters or “rare genomic changes” are relatively free of phylogenetic artifact [24,25,27], and therefore smaller numbers may be sufficient for robust phylogeny [33], it is clear that indels are far from free of homoplasy (Figure 7) [34,37]. Indels obviously can suffer from qualitative artifacts such as hidden paralogy, horizontal transfer and recombination [50], but they have also been shown to suffer from the quantitative phylogenetic artifacts of long branch attraction and taxon sampling effects [34,36,37]. However, in the absence of qualitative artifacts, CDIs can still be extremely useful as independent lines of evidence to test specific hypotheses [12,14,15,28,29] or additional characters to help improve resolution of substitution-based phylogenies [32].

### Patterns of insertion versus deletion

Singleton indels are the most easily interpreted indels, which makes them useful for examining general patterns of indel evolution. Such indels are particularly easy to identify here, because they are extracted from multiple sequence alignments. We find that singleton insertions occur at an equal or greater frequency than singleton deletions in every genome examined (Figure 9). This includes a wide range of evolutionary time scales, from roughly 10 to 1,000 million years (Figure 8) [51]. We also find that this ratio varies widely and with no apparent pattern across the tree (Figure 9). Some of the highest ratios are found in parasites, which have notoriously high evolutionary rates [28]. These include *Cryptococcus neoformans* (I:D = 7.5) and *Cyanidioschyzon merolae* (I:D = 6.0) (Figure 9). However, other parasitic species have relatively low ratios, such as *Entamoeba histolytica* (I:D = 1.47) and *Batrachochytrium dendrobatidis* (I:D = 2.0) (Figure 9). While there may be some variation in genome assembly quality among these taxa, the excess of insertions is consistent across the tree, making it unlikely that the overall I:D ratio is significantly affected by assembly errors in individual genomes.

One possible explanation of this strong and widespread insertion bias is a high background (neutral) bias toward DNA insertion across eukaryotes. However, indel rates in non-coding DNA seem to show a strong bias toward deletions, as well as a larger size for deletions compared to insertions [52]. For example, the rate of insertions versus deletions in *C. elegans* pseudogenes is 2.8 to 1 (I:D = 0.36) [53], which is very similar to the rates found in human pseudogenes (I:D = 0.33) [54]. On the other hand, mutation-accumulation lines of *C. elegans* show a slight insertion bias (I:D = 1.3), suggesting that pseudogenes may not be accurate indicators of neutral indel rates in coding sequences [53]. Nonetheless, although the insertion bias we find here may partly reflect a background bias toward insertions in DNA due to neutral processes, this is unlikely to explain the high average insertion bias we see (I:D = 2.31), much less the extremely high individual biases we find scattered across the tree (Figure 9).

Instead, we suggest that our results indicate that in-frame insertions in expansion regions of protein sequences experience less purifying selection than deletions. This may reflect the fact that deletions require removing established segments of protein sequence. Although these may have been neutral when first inserted, over time they may have acquired a function that contributes to their host’s fitness. Since protein indels mostly occur in external loops [6,22] and are more common in proteins that are highly inter-connected in protein interaction networks [3], insertions could provide opportunities for altering or fine-tuning intermolecular interactions [2]. Thus, insertions may initially serve as nearly neutral evolutionary experiments. The large variation in I:D rates seen here could, in part, indicate the relative importance of such processes in different lineages. Meanwhile the lowest rates (~1.0, Figure 9) may approach the neutral background rate as it is close to the neutral rate detected in *C. elegans* mutation accumulation lines (I:D = 1.31) [53]. Although this preference for insertions suggests that eukaryotic proteins should be increasing in size, this increase is quite small - the 550 singletons identified here are spread over 299 proteins and 35 taxa. Furthermore, many of these insertions may be ephemeral, *i.e*., easily reversed, particularly 1aa insertions [55]. Thus our results do not contradict the finding that protein size within eukaryotes is fairly stable [8].

## Conclusions

We find a substantial number of CDIs among major groups of eukaryotes, although these are unevenly distributed and mostly small (Figure 8). However, the number is too small and the level of homoplasy too high (Figure 7) to make it likely that phylogenetic analysis of indels alone can accurately reconstruct deep eukaryote branches. It is disappointing that large simple CDIs, the ideal class of phylogenetic indel and the easiest to identify, appear to be extremely rare in the most analytically tractable set of universal proteins. However, these proteins also harbor a large number of “slightly-complex” indels, among which some potentially useful CDIs might exist. For example, a large insertion in EF-1α first identified as a simple CDI exclusive to Metazoa and Fungi [10,12] is now known to exist in multiple variants. We are currently working on adapting SeqFIRE to identify such slightly-complex CDIs. Although the rather large, if highly variable insertion bias we identify here is surprising, given the fact that progressive sequence alignment methods tend to under-estimate insertions in multiple sequence alignments [56], it is likely that the 2.13 overall bias we find here is an under-estimate of the true rate of insertion bias in protein coding genes.

## Methods

### Proteomic sources

Thirty-five proteomes were selected to give a broad taxonomic sampling of three major groups of eukaryotes. These include proteomes from 16 Holozoa (1 choanoflagellate, 15 metazoa), eight Fungi, and nine Archaeplastida (1 red alga, 5 green algae, 3 land plants), plus two Amoebozoa. Sequences were downloaded as conceptual translations from the NCBI [57] and Joint Genome Institute (JGI) databases [58] (Additional file 2: Table S2).

### Identification of orthologous proteins

The protocol for orthologous protein identification and extraction is shown in Figures 1 and 2. An initial ortholog set was identified using the proteomes of *Danio rerio*, *Saccharomyces cerevisiae* and *Arabidopsis thaliana* as representatives of plants, animals and fungi, respectively. These proteomes were analyzed pairwise, and for each pairing all predicted proteins were grouped into putative orthologous clusters using InParanoid standalone version 3 [59]. The resulting pairwise clusters were then filtered and only the clusters with a single copy in both proteomes were retained. The three sets of single-copy putative orthologous clusters were then compared using a Python script, which identified 481 clusters with representatives from all three species. Clusters containing only short sequences (less than 250aa) were deleted, as these are usually too small to reliably assess orthology with molecular phylogeny. The result was 374 substantial universal, single-copy presumed orthologous clusters, which were then used as “seed orthologs”.

Orthologous sequences from 32 additional proteomes were obtained using all sequences in each cluster of seed orthologs as queries for standalone BLASTp searches against individual proteomes. A Python script was used to retrieve target sequences using three criteria. (i) If any E-value = 0.0 hits were found only these sequences were collected. (ii) If there were no E-value = 0.0 hits, all hits with an E-value < 1e-65 were extracted and further screened (see below). (iii) If no hits were found with E-value < 1e-65, the first hit with an E-value < 1e-30 was collected. The result was three match categories – (i) highly conserved, (ii) moderately conserved and (iii) poorly conserved, respectively. For category (ii), hits were further sorted by the difference in E-value (E-value distance) between individual hits and the top hit (|*d*_*x,y*_|) - for example, *d*_*1,2*_ is the E-value distance between the second and the first hit. A median was then calculated for the entire set of E-value distances and used as a cutoff, with the result that all hits with |*d*_*x*_*,*_*y*_| > cutoff were retained. All hits for each orthologous cluster were stored together in a single FastA file.

Each of the 374 FastA files was aligned using MUSCLE version 3.6 [40,41]. Then, all alignments were classified into 4 groups: (i) alignments with sequences from all taxa – “complete dataset” (5 alignments), (ii) alignments missing only a few sequences from species not located on a deep branch (major branch with only one possible representative in the full data set) – “nearly complete dataset” (125 alignments), (iii) alignments missing sequences from a number of taxa (between 3 to 7 taxa), but they still include the deep branches that may still provide some useful indels – “patchy dataset” (169 alignments), and (iv) alignments missing a lot of sequences including from species located on deep branches of any major group or missing taxa in a whole major group – “flawed dataset” (80 alignments). After 80 alignments in the fourth category were removed, each of the 299 remaining alignments was filtered to eliminate redundant or incomplete sequences (less than 50% of the average length of the alignment) using neighbor-joining (NJ) distance trees with bootstrapping (BP) using SeaView version 4.4.0 [60].

Clusters with multiple sequences from some taxa were treated as follows. For all in-paralogs, defined as strongly supported (>70% BP) clades of sequences from a single species, the sequence giving the shortest branch was retained, after controlling for partial sequences. For clusters containing out-paralogs, each clade (out-paralogous group) was separated into a new individual alignment increasing the number of the alignments to 353. These alignments were again analyzed for taxon representation and re-classified into the four categories described above. The final result was 299 orthologous protein alignments (categories 1–3 above), which were then re-aligned using MUSCLE before indel extraction.

### Indel extraction and analyses

The indel regions were extracted from the 299 individual alignments using standalone SeqFIRE, which uses consensus sequences for the identification and extraction of indels [39]. Before indel extraction, a similarity survey curve was constructed to determine the optimum sequence similarity level for the consensus sequences. This was done with multiple runs of SeqFIRE using similarity threshold levels from 25 – 100% in incremental steps of 5% for all alignments. All other SeqFIRE parameters were left at the default levels (inter-indel space = 3 and substitution group = “NONE”). All indels were extracted as separate indel files using parameters that were determined above. Indels were classified as either simple or complex depending on the number of indel states using a single Python script.

## Abbreviations

A: Arabidopsis thaliana; Aa: Amino acid(s); BP: Bootstrapping; CDI: Clade defining indel (phylogenetically informative); CUT: Chlorophyte + ulvophyte + trebouxiophyte; D: Danio rerio; I:D: Insertion:deletion ratio; MSA: Multiple sequence alignment; NJ: Neighbor joining; S: Saccharomyces cerevisiae.

## Competing interests

The authors declare that they have no competing interests.

## Authors’ contributions

Both authors made major contributions to conception and design of analyses, interpretation of data and writing the manuscript. SLB conceived the study, and PA performed all analyses and wrote the first draft of the manuscript. Both authors have read and approved the final manuscript.

## Supplementary Material

Additional file 1: Table S1 Number of matches and mismatches to consensus sequences for universally aligned positions in 299 universal single copy (in-paralog only) protein orthologs. The similarity threshold for the consensus sequence is 70%. Numbers in parentheses show percentage of matches and mismatches for each taxon group. Click here for file

Additional file 2: Table S2 List of proteomes used in this study. Click here for file
