# Supplementary material for: Evolution of protein indels in plants, animals and fungi
Source: BMC Evol Biol. 2013 Jul 4;13:140. doi: 10.1186/1471-2148-13-140 (PMC3706215; doi:10.1186/1471-2148-13-140)
Supplement: Additional file 1: Table S1 — Number of matches and mismatches to consensus sequences for universally aligned positions in 299 universal single copy (in-paralog only) protein orthologs. The similarity threshold for the consensus sequence is 70%. Numbers in parentheses show percentage of matches and mismatches for each taxon group. [file 1471-2148-13-140-S1.pdf]

Supplementary Table S2 Number of matches and mismatches to consensus sequences for universally aligned positions in 299 universal single copy (in-paralog only) protein orthologs. The similarity threshold for the consensus sequence is 70%. Numbers in parentheses show percentage of matches and mismatches for each taxon group.

Group of Organisms	Matches	Mismatches	Total (*N*)
Animals	65,677 (70.5%)	27,448 (29.5%)	93,125
Fungi	54,225 (58.2%)	38,900 (41.8%)	93,125
Plants	56,032 (60.2%)	37,093 (39.8%)	93,125
