# Supplementary material for: Evolution of protein indels in plants, animals and fungi
Source: BMC Evol Biol. 2013 Jul 4;13:140. doi: 10.1186/1471-2148-13-140 (PMC3706215; doi:10.1186/1471-2148-13-140)
Supplement: Additional file 2: Table S2 — List of proteomes used in this study. [file 1471-2148-13-140-S2.pdf]

Supplementary Table S1 List of proteomes used in this study.

Group	Proteomes	Source
Metazoa	*Homo sapiens*	NCBI
	Monodelphis domestica	NCBI
	Ornithorhynchus anatinus	NCBI
	Gallus gallus	NCBI
	Xenopus tropicalis	NCBI
	Danio rerio	NCBI
	Ciona intestinalis	NCBI
	Branchiostoma floridae	NCBI
	Strongylocentrotus purpuratus	NCBI
	Daphnia pulex	JGI
	Apis mellifera	NCBI
	Helobdella robusta	JGI
	Capitella sp.	NCBI
	Lottia gigantea	JGI
	Trichoplax adhaerens	NCBI
	Monosiga brevicollis	JGI
Fungi	*Saccharomyces cerevisiae*	NCBI
	Cochliobolus heterostrophus	JGI
	Aspergillus niger	JGI
	Aspergillus fumigatus	NCBI
	Cryptococcus neoformans	NCBI
	Laccaria bicolor	JGI
	Phycomyces blakesleeanus	JGI
	Batrachochytrium dendrobatidis	JGI
Amoebozoa	*Dictyostelium discoideum*	NCBI
	Entamoeba histolytica	NCBI
Viridieplantae	*Cyanidioschyzon merolae*	JGI
	Chlorella sp.	JGI
	Chlorella vulgaris	JGI
	Volvox carteri	JGI
	Chlamydomonas reinhardtii	JGI
	Ostercoccus lucimarinus	JGI
	Oryza sativa	JGI
	Arabidopsis thaliana	NCBI
	Physcomitrella patens	JGI
